# Cobalamin Deficiency: Effect on Homeostasis of Cultured Human Astrocytes

**DOI:** 10.3390/cells8121505

**Published:** 2019-11-24

**Authors:** Zuzanna Rzepka, Jakub Rok, Michalina Respondek, Justyna Pawlik, Artur Beberok, Dorota Gryko, Dorota Wrześniok

**Affiliations:** 1 Department of Pharmaceutical Chemistry, Faculty of Pharmaceutical Sciences in Sosnowiec, Medical University of Silesia, Katowice, Poland, Jagiellońska 4, 41-200 Sosnowiec, Poland; zrzepka@sum.edu.pl (Z.R.); jrok@sum.edu.pl (J.R.); mrespondek@sum.edu.pl (M.R.); j.pawlik@med.sum.edu.pl (J.P.); 2 Institute of Organic Chemistry, Polish Academy of Science, Kasprzaka 44/52, 01-224 Warsaw, Poland; dorota.gryko@icho.edu.pl

**Keywords:** cobalamin, vitamin B_12_ deficiency, neurological disorders, astrocytes

## Abstract

Cobalamin deficiency is an important health problem. The major non-hematological symptoms of hypocobalaminemia are nervous system disorders, but the molecular and cellular mechanisms underlying this phenomenon have not yet been fully explained. Increasing scientific evidence is stressing the pivotal role of astrocyte dysfunction in the pathogenesis of a wide range of neurological disorders. In light of the above, the aim of this study was to develop an in vitro model of cobalamin deficiency by optimizing the conditions of astrocyte culture in the presence of vitamin B_12_ antagonist, and then the model was used for multidirectional analysis of astrocyte homeostasis using image cytometry, immunoenzymatic and colorimetric assays, and fluorescence spectroscopy. Our results indicated that long-term incubation of normal human astrocytes with hydroxycobalamin(*c*-lactam) causes an increase of extracellular homocysteine level, a reduction of cell proliferation, and an accumulation of cells in the G_2_/M cell cycle phase. Moreover, we observed dramatic activation of caspases and an increase of catalase activity. Interestingly, we excluded extensive apoptosis and oxidative stress. The study provided significant evidence for astrocyte homeostasis disturbance under hypocobalaminemia, thus indicating an important element of the molecular mechanism of nervous system diseases related to vitamin B_12_ deficiency.

## 1. Introduction

Cobalamin (vitamin B_12_) deficiency is an important global public health problem. It can affect people of all ages, but it most particularly affects pregnant women and the elderly. The deficiency is caused by either inadequate intake (when animal-derived foods are restricted), decreased bioavailability or malabsorption, as well as genetically determined disruption of vitamin B_12_ transport in the blood, cellular uptake, or metabolic pathway [1,2,3]. Moreover, there are up-to-date pharmacotherapy-related risk factors for cobalamin deficiency, namely, prolonged use of metformin [4] and proton pump inhibitors [5].

Vitamin B_12_ deficiency express itself by a wide variety of hematological, neurological, psychiatric, gastrointestinal, and skin disorders [6,7]. Magaloblastic anemia is highly characteristic and is therefore a primary indicator in the diagnosis of a hypocobalaminemia. However, nervous system disorders, such as brain atrophy, myelopathy, and neuropathy, are often the earliest and, in some cases, the only clinical symptoms of vitamin B_12_ deficiency [6,8,9].

Cobalamin has an important role in cellular functioning, especially in DNA synthesis, methylation, and mitochondrial metabolism [1]. Its depletion may result in genome instability and an accumulation of methylmalonic acid and homocysteine (Hcy), which have prooxidant and cytotoxic activity [10,11,12]. Much is now known about the biochemistry of vitamin B_12_; however, the effect of its deficiency on homeostasis of particular cell types is still unclear. This is due to the difficulty of obtaining a suitable experimental model. It has been demonstrated that the use of the vitamin B_12_ antagonist hydroxycobalamin(*c*-lactam), abbreviated as (OH)Cbl(*c*-lactam), may be an effective method of inducing hypocobalaminemia in cellulo [13]. Nonetheless, to obtain the powerful experimental model, the conditions of incubation with the agent must be optimized and adapted to a specific cell type.

In the nervous system, glia, myelin sheaths, and the interstitium are the components mainly affected by cobalamin deficiency [14]. Astrocytes are known as glial cells that play many housekeeping functions in the nervous system, including structural, trophic, and metabolic support for neurons, maintenance of the extracellular environment, and redox homeostasis, as well as neurotransmitter synthesis and modulation of synaptic transmission [15]. Moreover, astrocytes respond to diverse forms of brain injury with heterogeneous functional and morphological changes that are collectively referred to as reactive astrogliosis [16]. There is a growing support for the concept of astrocytopathies in which the disruption of astrocyte homeostasis, astrodegeneration, or dysfunctional astrogliosis are the primary causes or the importants factor in neurological disorders [17]. Recent studies have revealed the link between astrocytes and some nervous system diseases such as Alzheimer’s disease, Parkinson’s disease, amyotrophic lateral sclerosis, and epilepsy through mechanisms that include oxidative stress, cell signaling, necrosis, or apoptosis [15,17,18]. The role of astrocytes in the pathogenic mechanism of neuropathy due to hypocobalaminemia has also been suggested [15]. In various experimental animal models of hypocobalaminemia, as well as in the central nervous system of deceased patients with neuropathy from vitamin B_12_ deficiency, an astrogliosis has been detected [14,19].

The aim of the present study was (i) to develop an in vitro model of cobalamin deficiency by culturing astrocytes in the presence of (OH)Cbl(*c*-lactam) until the functional deficiency reflected by the accumulation of homocysteine (marker of hypocobalaminemia) in the culture medium was observed; and (ii) to get general insight into the effect of vitamin B_12_ deficiency on homeostasis of cultured astrocytes. Considering the biochemical role of cobalamin and thus the likely effects of its deficiency at the cellular level, in the latter step, specific assays were performed to assess the cell cycle progression, apoptotic/necrotic/health cells ratio, caspase activation, and redox homeostasis.

## 2. Materials and Methods

### 2.1. Chemicals

5-(and 6)-chloromethyl-2′,7′-dichlorodihydrofluorescein diacetate (CM-H_2_DCFDA); Dulbecco’s phosphate-buffered saline (DPBS); diamidino-2-phenylindole (DAPI) solution (1 mg/mL); Halt Protease Inhibitor Cocktail; Halt Phosphatase Inhibitor Single-Use Cocktail; Gibco Astrocyte Medium consisting of Dulbecco’s modified Eagle’s medium (DMEM), N-2 Supplement, and One Shot fetal bovine serum (FBS); Pierce BCA Protein Assay Kit; and StemPro Accutase Cell Dissociation Reagent were purchased from Thermo Fisher Scientific (USA). Solution 3 (DAPI 1 µg/mL, triton X-100 0.1%), Solution 5 (VitaBright-48 400 µg/mL, propidium iodide 500 µg/mL, acridine orange 1.2 µg/mL), Solution 7 (JC-1 dye 200 µg/mL), Solution 8 (DAPI 1 µg/mL), Solution 15 (Hoechst 33,342 500 µg/mL), Solution 16 (propidium iodide 500 µg/mL), Via1-Cassettes, and NC-Slides A2 and A8 were obtained from ChemoMetec (Denmark). Caspase 3/7 Assay Kit, Caspase 8 Assay Kit, and Caspase 9 Assay Kit were purchased from Sigma-Aldrich (Germany) or ImmunoChemistry Technologies (USA). Superoxide Dismutase Assay Kit, Catalase Assay Kit, and Glutathione Peroxidase Assay Kit were obtained from Cayman Chemical (USA). Phalloidin-Atto565, penicillin G, and amphotericin B were purchased from Sigma-Aldrich (Germany). Annexin V-CF488A and Annexin V binding buffer were obtained from Biotium (USA). Neomycin sulfate was obtained from Amara (Poland). Human Hcy ELISA Kit was obtained from Abbexa (United Kingdom). The remaining chemicals were purchased from POCH S.A. (Poland) or Sigma-Aldrich (Germany). Hydroxycobalamin(*c*-lactam) was synthesized by Prof. Dorota Gryko and Dr. Keith ó Proinsias (Institute of Organic Chemistry, Polish Academy of Science, Warsaw, Poland). The synthesis and identification of the compound were performed as described previously [13].

### 2.2. Cell Culture

Gibco Human Astrocytes (Thermo Fisher Scientific) were cultured according to the manufacturer’s instructions. Cells were maintained in a humidified 5% CO_2_ incubator at 37 °C. Growth medium consisted of Dulbecco’s modified Eagle’s medium (DMEM), N-2 Supplement, One Shot fetal bovine serum (FBS), penicillin G (100 U/mL), neomycin (10 μg/mL), and amphotericin B (0.25 μg/mL). The experiments were performed using cells from passage 4–9.

### 2.3. The Induction Of Cobalamin Deficiency

To achieve an experimental model of cobalamin deficiency, after preliminary experiments under different cell culture conditions (e.g., density of the cells, concentration of the agent), astrocytes were seeded into a T-25 flask (100,000 cells/flask) and cultured in medium supplemented with (OH)Cbl(*c*-lactam) at a concentration of 20 μg/mL. Control culture (astrocytes incubated in standard growth medium) was cultivated in parallel. At days 7, 13, 20, and 27, culture medium was collected and centrifuged at 2000× *g* for 20 min and the supernatants were aliquoted and stored at 20 °C until further analysis, that is, Hcy concentration measurements. At indicated days, cells were passaged to 100,000 cells/flask and the experiment was terminated on day 27 due to persistent inhibition of cell growth in the treated culture.

### 2.4. Cell Count Assay

Astrocytes were counted using the image cytometer NucleoCunter NC-3000 controlled by the NucleoView NC-3000 Software (ChemoMetec). In brief, cells were detached with accutase and the samples of obtained cell suspensions were loaded into the Via1-Cassette (ChemoMetec) containing acridine orange and DAPI.

### 2.5. Analysis of Extracellular Homocysteine Level

The cellular metabolic disorders due to vitamin B_12_ deficiency result in the accumulation of Hcy and its export to culture medium [20]. To verify whether the astrocytes cultured with (OH)Cbl(*c*-lactam) were cobalamin deficient, concentration of Hcy was measured in the medium samples collected at days 7, 13, 20, and 27. For this purpose quantitative sandwich enzyme-linked immuno-sorbent assay (ELISA) was performed using commercially available Human Hcy ELISA Kit (Abbexa), according to the previously described method [13].

### 2.6. Cell Cycle Assay and DNA Fragmentation Assay

Cell cycle phase distribution of astrocytes after the 27 day culture in medium with/without (OH)Cbl(*c*-lactam) was analyzed following a previously described method [21]. In brief, cells were fixed with ice-cold 70% ethanol at 4 °C for 24 h, stained with Solution 3 for 5 min at 37 °C, and then analyzed using the image cytometer with the adequate protocol—Cell Cycle Analysis of Fixed Cells or DNA Fragmentation Assay (ChemoMetec).

### 2.7. Mitochondrial Potential Assay

Astrocytes at day 27 of culture with/without (OH)Cbl(*c*-lactam) were examined in terms of the mitochondrial transmembrane potential (ΔΨ_m_). The analysis was performed using the image cytometer NucleoCounter NC-3000 following JC-1/DAPI staining according to a previously described method [21].

### 2.8. Annexin V Assay

Phosphatidylserine externalization in astrocytes after the 27 day culture in medium with/without (OH)Cbl(*c*-lactam) was detected by a previously described method [22] using the image cytometer and CF488A-labeled annexin V.

### 2.9. Caspase Activity Assay

The activity of caspase 3/7, 8, and 9 in astrocytes after 27 days of culture in medium with/without (OH)Cbl(*c*-lactam) was assessed using the image cytometer and the fluorescent inhibitor of caspases (FLICA reagents) containing carboxyfluorescein (FAM). The analysis was performed separately for caspase 3/7, caspase 8, and caspase 9. The respective FLICA reagent and Hoechst 33,342 were added to the cell suspensions and incubated for 60 min at 37 °C. After incubation, cell pellets were washed, then resuspended in apoptosis wash buffer supplemented with propidium iodide and analyzed immediately.

### 2.10. Assessment of Cell Morphology

Astrocytes treated with (OH)Cbl(*c*-lactam) for 27 days and control astrocytes were seeded (50,000 cells/dish) on separate sterile cover slips placed in Petri dishes and were allowed to attach for 24 h. Following fixation with paraformaldehyde (4%), cells were stained with DAPI (3 µg/mL) and Phalloidin–Atto 565 (0.6 µM) to visualize cell nucleus and actin filaments, respectively. The cover slips were mounted onto a microscopic glass slide. The samples were scanned using a Nikon A1R Si confocal imaging system with a Nikon Eclipse Ti-E inverted microscope controlled by Nikon NIS Elements AR software.

### 2.11. Antioxidant Enzyme Activity Assays

Intracellular activity of superoxide dismutase (SOD), catalase (CAT), and glutathione peroxidase (GPx) were estimated using the spectrophotometric assay kits (Cayman) according to a previously described method [23]. The analyses were performed on cell lysates prepared from astrocytes at day 27 of the culture in medium with/without (OH)Cbl(*c*-lactam). The absorbance measurements were carried out using the microplate reader Infinite 200 Pro controlled by the Magellan software. Cellular protein concentration was determined by the use of the Pierce BCA Protein Assay Kit (Thermo Fisher Scientific) and microvolume spectrophotometer DeNovix DS-11. The measurements were performed using the microplate reader Infinite 200 Pro controlled by i-control software (Tecan). The enzyme activity in the treated astrocytes was expressed as percentage of the control cells.

### 2.12. Intracellular ROS Level Assay

Reactive oxygen species (ROS) levels in astrocytes were estimated using the cell-permeable non-fluorescent probe CM-H_2_DCFDA. After intracellular deacetylation, ROS oxidize the probe to generate a highly fluorescent product. The fluorescence intensity is correlated with the intracellular ROS level [24]. At day 27 of the culture in medium with/without (OH)Cbl(*c*-lactam), cells were seeded at a density of 5 × 10^3^ cells per well into 96-well clear bottom black microplates. Following 48 h of incubation, cells were treated with CM-H_2_DCFDA (working concentration: 5 μM) in the dark for 30 min and washed twice with DPBS. The fluorescence intensity (λ_ex_ = 495 nm, λ_em_ = 527 nm) was measured using the microplate reader Infinite 200 Pro controlled by the Magellan software. The results obtained for treated astrocytes were expressed as percentage of the control cells.

### 2.13. Intracellular Thiol Status

Intracellular thiols, by their ability to be reversible oxidized, determine cellular redox homeostasis, and thus the decrease of reduced thiol level is recognized as a marker of oxidative stress [25]. Analysis of thiol status in astrocytes after the 27 day culture in medium with/without (OH)Cbl(*c*-lactam) was performed using the NucleoCounter NC-3000 system according to a previously reported method [13]. In brief, the assay is based on staining with a VitaBright 48 (VB-48) probe, which selectively reacts with free thiol groups forming a fluorescent product [26].

### 2.14. Statistical Analysis

Data are presented as mean values ± SD of three independent experiments in at least three repetitions. Differences between groups were analyzed by unpaired *t*-test or one-way ANOVA followed by Tukey’s post-hoc test, as appropriate, using GraphPad Prism 8.0.1 software. *p*-value < 0.05 was considered indicative of a statistically significant difference.

## 3. Results

### 3.1. Effect of Long-Term Treatment With (OH)Cbl(c-lactam) on Astrocyte Proliferation and Extracellular Hcy Level

**Figure 1:**
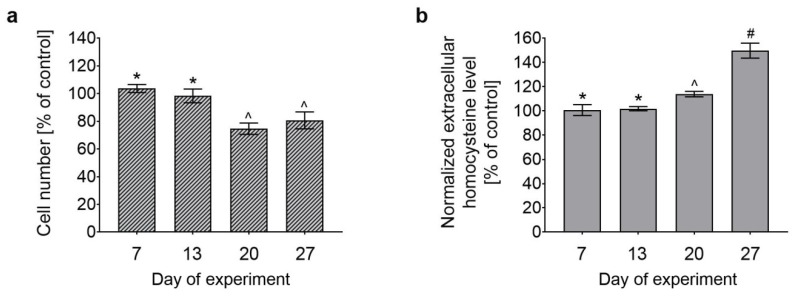
Effect of long-term treatment with (OH)Cbl(*c*-lactam) on astrocyte proliferation and extracellular homocystine level. Normal human astrocytes were cultured in medium supplemented with (OH)Cbl(*c*-lactam) in concentration of 20 μg/mL for 27 days. Control cells were cultured in parallel in standard growth medium. (**a**) Total cell number determined at days 7, 13, 20, and 27. (**b**) Homocysteine concentration in medium collected at the indicated time points—the results were normalized to 100,000 cells. All data were expressed as a percentage of control and presented as the mean values ± SD of three independent experiments (where the analysis was performed in at least three replicates); data not sharing common symbols were different from each other, *p* < 0.05 (* for controls; one-way ANOVA followed by Tukey’s test).

Normal human astrocytes were cultured in growth medium supplemented with (OH)Cbl(*c*-lactam) at a concentration of 20 μg/mL for 27 days. During subculturing, cell count was determined using NucleoCounter NC-3000, as described in Section 2.4. As demonstrated in Figure 1a, at day 20 and 27 of the treatment, the number of cells was significantly lower than in the control culture.

The state of hypocobalaminemia in cellulo can be detected by measuring extracellular homocysteine level [13]. To determine a time after which astrocytes cultured with (OH)Cbl(*c*-lactam) were cobalamin deficient, we measured the concentration of homocysteine in the medium samples collected during cell subculturing. We observed that at day 20 and 27 of the treatment, the level of the biomarker was 114% and 150% of the control, respectively (Figure 1b).

The observed increase in the extracellular homocysteine level (Figure 1b) indicated that cobalamin deficiency was successfully induced in human astrocytes after 27 days of culture with (OH)Cbl(*c*-lactam) in a concentration of 20 μg/mL. Thus, we obtained an in vitro model for investigation whether/how hypocobalaminemia may affect homeostasis of astrocytes.

Because we observed an inhibitory effect of vitamin B_12_ deficiency on astrocyte proliferation (Figure 1a), the consequent step was to characterize whether this property was exerted by arrest in cell cycle, apoptosis, or both.

### 3.2. Cell Cycle of Astrocytes Under Conditions of Cobalamin Deficiency

**Figure 2:**
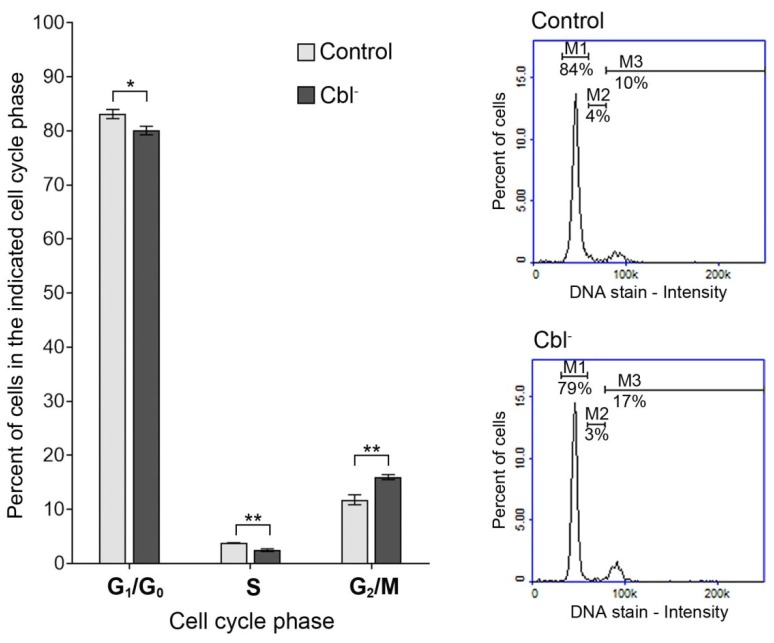
Effect of cobalamin deficiency on cell cycle in cultured astrocytes. Cell cycle analysis was performed for cobalamin deficient (Cbl^-^) and control astrocytes. Bar graph represents mean values ± SD of three independent experiments (where the analysis was performed in at least three replicates); * *p* < 0.05, ** *p* < 0.01 (unpaired *t*-test). The presented histograms are representative of three independent experiments: M1—cells in G_1_/G_0_ phase, M2—cells in S phase, M3—cells in G_2_/M phase.

Cell cycle analysis by DNA content measurement was performed by the use of the fluorescence image cytometer. The obtained data revealed that within the cobalamin-deficient population (cells treated with the antagonist for 27 days) there was a reduction of cell number in G_1_/G_0_ and S phase and a corresponding increase in G_2_/M phase, compared with the control cells (Figure 2).

### 3.3. Evaluation of Apoptotic Markers in the Cobalamin-Deficient Astrocytes

Apoptosis is a form of cell death characterized by several features, including mitochondrial membrane depolarization, phosphatidylserine externalization, and DNA fragmentation [27]. Due to its implication in the pathogenesis of neurodegenerative disorders, apoptosis is intensively studied in various experimental models involving nervous system cells.

#### 3.3.1. Mitochondrial Membrane Depolarization

**Figure 3:**
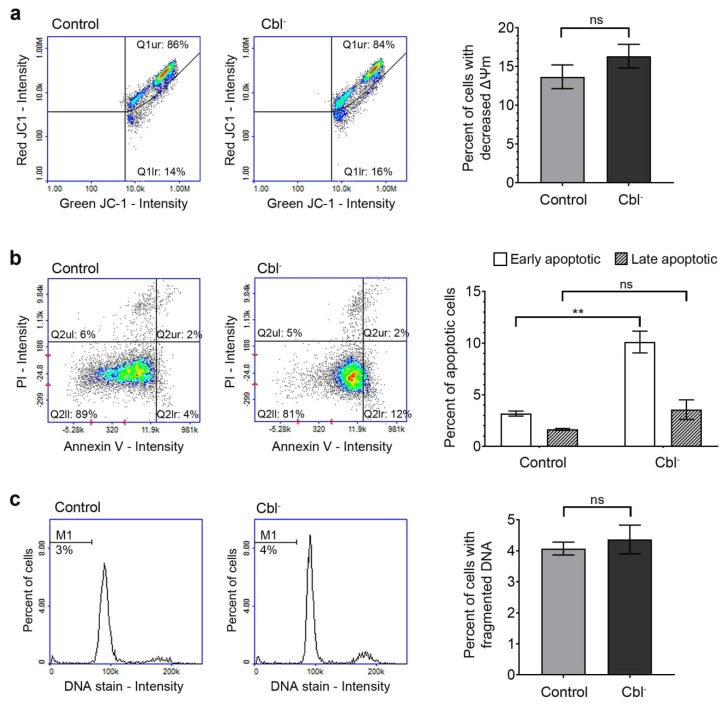
Evaluation of apoptosis induction by cobalamin deficiency in cultured astrocytes. The assays were performed for cobalamin deficient (Cbl^-^) and control astrocytes. (**a**) Mitochondrial membrane potential determined following staining of cells with JC-1 dye. (**b**) Phoshatidylserine externalization detected using fluorescently labeled annexin V probe. (**c**) DNA fragmentation evaluated by the measurement of cellular DNA content. The presented scatter plots and histograms are representative of three independent experiments: Q1ur—cells with polarized mitochondria, Q1lr—cells with depolarized mitochondria, Q2ul—necrotic cells, Q2ur—late apoptotic cells, Q2ll—healthy cells, Q2lr—early apoptotic cells, M1—cells with fragmented DNA. Bar graphs represent mean values ± SD of three independent experiments (where the analysis was performed in at least three replicates); ** *p* < 0.01 (unpaired *t*-test).

Mitochondrial membrane potential is a parameter of mitochondria function that is used as an indicator of apoptosis [28]. The effect of cobalamin deficiency on mitochondrial membrane potential was evaluated according to the method described in Section 2.7. Analysis of the results obtained for control and cobalamin-deficient astrocytes (Figure 3a) revealed that cobalamin deficiency was not associated with mitochondrial membrane depolarization in the treated cells.

#### 3.3.2. Phosphatidylserine Externalization

Translocation of phosphatidylserine from the inner to the outer membrane layer occurs early in apoptosis and may be detected using fluorescently labeled annexin V [29]. As the membrane integrity of late apoptotic cells is lost, they may be distinguished from early apoptotic cells using staining with propidium iodide (PI). As presented in Figure 3b, we observed slight difference between the percentage of early apoptotic (annexin V-positive/PI-negative) cells in the population of control (3.2% ± 0.2%) and cobalamin-deficient astrocytes (10.1% ± 1.1%). However, there was no significant difference in the percentage of late apoptotic (annexin V-positive/PI-positive) cells between compared groups.

#### 3.3.3. DNA Fragmentation

DNA fragmentation, the end stage of apoptosis, was evaluated using fluorescence image cytometer. As presented in Figure 3c, no induction of DNA fragmentation due to cobalamin deficiency was found in the studied astrocyte-based model. The results were consistent with the results from the annexin V assay, which indicated the lack of late apoptosis in (OH)Cbl(*c*-lactam)-treated astrocytes.

### 3.4. Caspase Activation in the Cobalamin-Deficient Astrocytes

**Figure 4:**
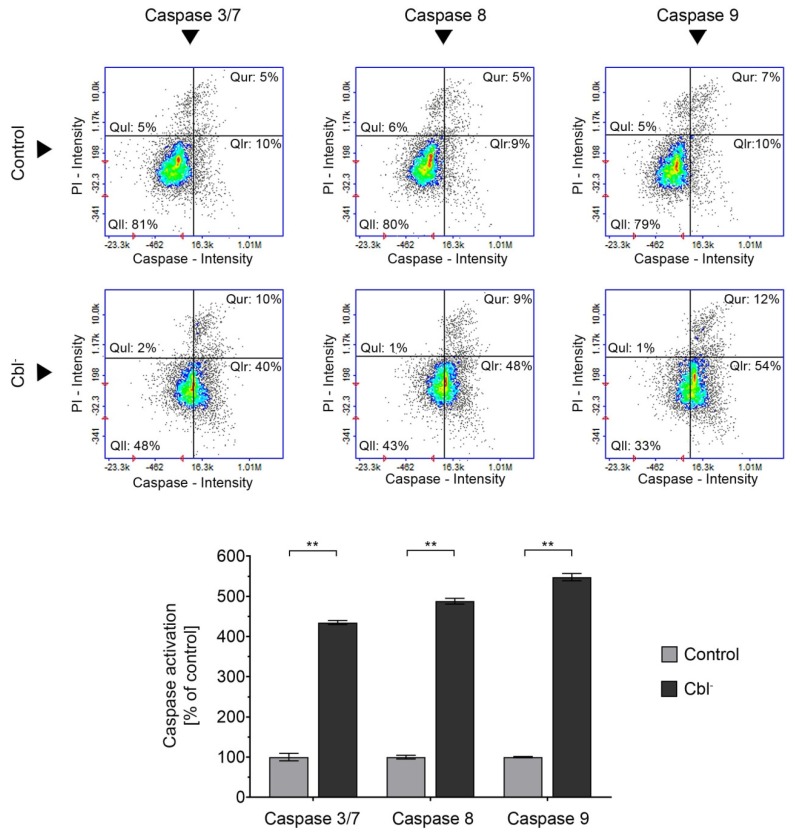
Effect of cobalamin deficiency on caspase activation in astrocytes. The assays were performed for cobalamin deficient (Cbl^-^) and control astrocytes. The presented scatter plots are representative of three independent experiments: Qul—propidium iodide (PI)-positive cells with non-activated caspase, Qll—living cells with non-activated caspase, Qur—PI-positive cells with activated caspase, and Qlr—living cells with activated caspase. Bar graph represents mean values ± SD of three independent experiments (where the analysis was performed in at least three replicates); ** *p* < 0.01 (unpaired *t*-test).

Although caspases, a family of cysteine proteases, are mostly known as executioners of apoptosis, recent studies have indicated that these enzymes also control various nonlethal cellular activities such as proliferation, differentiation, or cytoskeleton reorganization [30] and, in case of astrocytes, astrogliosis [31,32]. In the current study, we examined the activation of caspase 3/7, 8, and 9 by the use of fluorescence image cytometer. Our results indicatef that cobalamin deficiency caused a dramatic increase of caspase activity—by 335%, 388%, or 448% for caspase 3/7, 8, or 9, respectively (all compared to the control; Figure 4). Considering the results presented in Figure 3, which indicate the absence of extensive induction of apoptosis, we observed that cobalamin deficiency may result in non-apoptotic caspase activation in astrocytes.

### 3.5. Effect of Cobalamin Deficiency on Astrocyte Morphology

**Figure 5:**
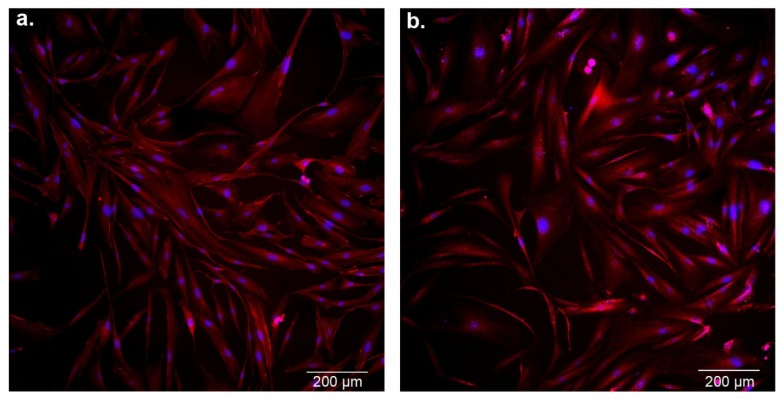
Confocal microscopic images of control astrocytes (**a**) and cobalamin-deficient asctrocytes (**b**). Cells on coverslips were fixed and labeled with Atto 565-conjugated phalloidin for actin visualization (red channel), whereas nuclei were labeled with diamidino-2-phenylindole (DAPI; blue channel). Scale bar = 200 µm.

The morphology of astrocytes was estimated by the use of confocal microscopy (Figure 5). The untreated (control) and cobalamin-deficient cells showed all the original properties such as adherent growth, regular shape, and cell–cell cohesion. No morphological features of apoptosis, such as cell shrinkage, membrane blebbing, cytoplasmic condensation, and formation of membrane-bound apoptotic bodies, were observed in the analyzed cells.

### 3.6. Redox Homeostasis of Astrocytes under Conditions of Cobalamin Deficiency

**Figure 6:**
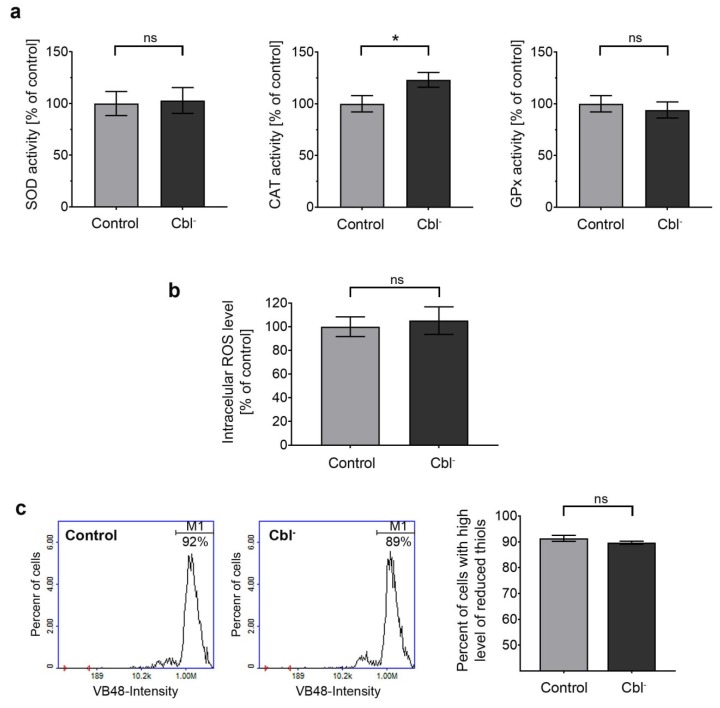
Effect of cobalamin deficiency on redox homeostasis in cultured astrocytes. The analyses were performed for cobalamin-deficient (Cbl^-^) and control astrocytes. (**a**) Superoxide dismutase (SOD), catalase (CAT), and glutathione peroxidase (GPx) activity was determined using specific colorimetric assays. (**b**) Intracellular reactive oxygen species (ROS) level evaluated by the use of CM-H_2_DCFDA probe. (**c**) Reduced thiol level determined following the staining of cells with VitaBright 48 (VB-48); the presented histograms are representative of three independent experiments; M1—cells with high level of reduced thiols. Bar graphs represent mean values ± SD of three independent experiments where the analysis was performed in at least three replicates; * *p* < 0.05 (unpaired *t*-test).

Under physiological conditions, cells are in a stable state known as redox homeostasis, which is maintained by the balance between reactive oxygen species generation and cellular antioxidant efficiency [33]. To determine whether hypocobalaminemia may affect redox homeostasis in astrocytes, we examined (i) activity of the antioxidant enzymes (superoxide dismutase, catalase, and glutathione peroxidase), (ii) intracellular ROS level, and (iii) reduced thiol status in cells treated with (OH)Cbl(*c*-lactam) for 27 days. No significant differences were observed between cobalamin-deficient and control cells, except for a catalase assay in which a slight increase (by 23%) in the enzyme activity was noted (Figure 6a).

## 4. Discussion

Vitamin B_12_ deficiency has great clinical relevance as it may be linked to severe or even life-threatening disorders [8]. Hypocobalaminemia particularly affects bone marrow and nervous systems. The neuropsychiatric manifestations of hypocobalaminemia show a wide range of variation and include paraesthesias, skin numbness, coordination disorders, paraparesis or tetraparesis, dementia, confusion, stupor, apathy, psychosis, and depression [6,8]. The molecular and cellular mechanism of these symptoms is still unknown. Taking into account the fact that a disturbance of astrocytes homeostasis was shown to play a role in patomechanism of various neurological and mental disorders [17], in the current study we investigated, for the first time, the multifaceted impact of cobalamin deficiency on astrocytes in vitro.

Cobalamin analogues with a modification of the amide group present at the *c*-position of B pyrrolic ring [34] were demonstrated to be an efficient antagonist of the vitamin because the use of these agents in various biological systems (experimental animals, cell lines) resulted in an inhibition of cobalamin-dependent enzymes (methionine synthase and methylmalonyl-CoA mutase) [7,13,35,36,37]. Thus, the agents were used in several studies to induce the state of cobalamin deficiency in experimental conditions, both in vivo [7] and in vitro, for example, in the culture of human leukemia cells [35], rat oligodendrocytes [36], and human proximal tubule cells [37]. Previously, we developed an experimental in vitro model of hypocobalaminemia in normal human melanocytes by treating the cells with (OH)Cbl(*c*-lactam) in a concentration of 10 µg/mL for 24 days [13]. Here, we described the astrocyte-based model assuming 27 day culture in medium supplemented with the cobalamin antagonist in one-fold higher concentration. Significant increase of extracellular homocysteine level and concomitant inhibition of cell proliferation (Figure 1) were recognized as indicators of vitamin B_12_ deficiency. The aim of our study was to gain a model based on viable cells, and thus the extensive apoptosis/necrosis was not desirable. On purpose, we used the agent in concentration and time of treatment that was not toxic itself to astrocytes (Figure 3) but sufficient enough to induce the state of cobalamin deficiency.

In order to explore the antiproliferation effect of cobalamin depletion in astrocytes, cell cycle distribution of control and (OH)Cbl(*c*-lactam)-treated cells was analyzed by image cytometry. In the latter cell population, there was a slight reduction of cells in G_1_/G_0_ and S phase and a corresponding increase in G_2_/M phase (Figure 2), which may suggest that cobalamin depletion in astrocytes induces disorders of DNA synthesis. Studies on cells from patients with megaloblastic anemia indicated that cobalamin and/or folate deficiency suppress DNA synthesis, and that when DNA synthesis is impaired, the cell cycle cannot progress from the G_2_ phase to mitosis, leading to cell growth without division, which presents as macrocytosis [38,39]. Moreover, Huang et al. [40] revealed that folate deficiency in HepG2 cells resulted, apart from apoptosis, in an accumulation of the cells in S and G_2_/M phase of cell cycle.

Astrocytes outnumber neurons in the brain and play many roles essential for normal function of the nervous system, including participation in neuronal metabolism, synaptic transmission, blood–brain barrier formation, and neuroprotection. It was demonstrated that astrocytes respond to central nervous system injury with a reactive state, referred to as astrogliosis, which is characterized by some morphological and functional changes [41]. Studies performed by Guyenet et al. [31] and Aras et al. [32] revealed that astrogliosis is associated with non-apoptotic activation of caspases. This finding may be crucial for explaining the results presented in our study—the dramatic increase in caspase 3/7, 8, and 9 activity (Figure 4) without the concomitant extensive apoptotic effect, as confirmed by the negative results of mitochondrial membrane depolarization, DNA fragmentation, and phosphatidylserine externalization assays (Figure 3), as well as microscopic observation (Figure 5). However, further specific studies are needed to confirm this hypothesis.

Features of astrogliosis have been observed in the astrocytes from the spinal cord of rats with cobalamin deficiency induced by means of total gastrectomy or a chronic cobalamin-depleted diet [14]. Astrocytes are the main cells in the nervous system that produce and release cytokines and growth factors. Scalabrino et al. described how in rat spinal cord, vitamin B_12_ deficiency increases the synthesis of myelinotoxic cytokines (e.g., tumor necrosis factor) and a myelinotoxic growth factor (nerve growth factor), but decreases synthesis of a myelinotrophic cytokine (interleukin-6) and a myelinotrophic growth factor (epidermal growth factor). They suggested that the imbalance of cytokines and growth factors may be essential to the pathogenesis of the white matter lesions and thus neuropathy due to cobalamin deficiency [14,19].

The body of scientific evidence supports the role of cobalamin as a modulator of redox homeostasis and the relationship between cobalamin deficiency and oxidative stress [42]. We performed the analysis of the activity of major cellular antioxidant enzymes—SOD, CAT, and GPx. Our results indicated that cobalamin deficiency led to the increase of catalase activity in astrocytes, whereas the activity of SOD and GPx was not affected in the treated cells (Figure 6a). According to Desagher et al. [43], catalase is crucial for the neuroprotective effect of astrocytes. In the current study, we observed no effect of cobalamin deficiency on intracellular ROS level and thiol status (Figure 6b,c). In contrast, previous studies on human melanocytes [13] and human proximal tubule cells [37] revealed that cobalamin deficiency may cause depletion of reduced thiol pools. We hypothesized that this inconsistency may result from different susceptibility to prooxidant conditions depending on the type of cells examined.

## 5. Conclusions

In conclusion, we demonstrated that culturing of human astrocytes with (OH)Cbl(*c*-lactam) in a concentration of 20 μg/mL for 27 days provided an effective in vitro model for investigation on whether/how hypocobalaminemia can affect astrocytes. Taken together, the obtained results revealed that hypocobalaminemia impaired astrocyte homeostasis, which may be one of the mechanisms underlying nervous system disorders and the neurodegeneration process related to vitamin B_12_ deficiency.
